# Comparison of *E. coli* based self-inducible expression systems containing different human heat shock proteins

**DOI:** 10.1038/s41598-021-84188-8

**Published:** 2021-02-25

**Authors:** Fatemeh Sadat Shariati, Malihe Keramati, Vahideh Valizadeh, Reza Ahangari Cohan, Dariush Norouzian

**Affiliations:** Department of Nanobiotechnology, New Technologies Research Group, Pasteur Institute of Iran, Tehran, Iran

**Keywords:** Biological techniques, Biotechnology, Developmental biology, Microbiology, Molecular biology

## Abstract

IPTG-inducible promoter is popularly used for the expression of recombinant proteins. However, it is not suitable at the industrial scale due to the high cost and toxicity on the producing cells. Recently, a Self-Inducible Expression (SILEX) system has developed to bypass such problems using Hsp70 as an autoinducer. Herein, the effect of other heat shock proteins on the autoinduction of green fluorescent protein (EGFP), romiplostim, and interleukin-2 was investigated. For quantitative measurements, EGFP expression was monitored after double-transformation of pET28a-EGFP and pET21a-(Hsp27/Hsp40/Hsp70) plasmids into *E.* *coli* using fluorimetry. Moreover, the expression level, bacterial growth curve, and plasmid and expression stability were compared to an IPTG- inducible system using EGFP. Statistical analysis revealed a significant difference in EGFP expression between autoinducible and IPTG-inducible systems. The expression level was higher in Hsp27 system than Hsp70/Hsp40 systems. However, the highest amount of expression was observed for the inducible system. IPTG-inducible and Hsp70 systems showed more lag-time in the bacterial growth curve than Hsp27/Hsp40 systems. A relatively stable EGFP expression was observed in SILEX systems after several freeze–thaw cycles within 90 days, while, IPTG-inducible system showed a decreasing trend compared to the newly transformed bacteria. Moreover, the inducible system showed more variation in the EGFP expression among different clones than clones obtained by SILEX systems. All designed SILEX systems successfully self-induced the expression of protein models. In conclusion, Hsp27 system could be considered as a suitable autoinducible system for protein expression due to less metabolic burden, lower variation in the expression level, suitable plasmid and expression stability, and a higher expression level.

## Introduction

*Escherichia coli* is a widely used expression host for the production of recombinant proteins in commercial and research fields because of easy manipulation and low-cost production. However, overexpression of a recombinant protein by the host often imposes a high metabolic burden that requires critical optimization of cell density, inducer concentration, and expression monitoring^1,2^. To date, isopropyl-β-D-1-thiogalactopyranoside (IPTG) is the most popular inducer in control of the expression level of recombinant proteins in lac promoter-based plasmids. In addition to IPTG-dependent promoters (T7 promoters), different inducible promoters are being continually developed that are induced by lactose^3,4^, galactose^5^, L-arabinose^6^, glycerol, tetracycline^7^, crystal violet dyes^8^, and uric acid^9^. However, in all cases, the use of an inducer deals with technical difficulties, particularly at the industrial scale. Using inducers not only imposes extra costs for production but also associates with practical issues such as cell density monitoring and determination of optimum induction point which is usually variable among recombinant hosts. Moreover, the expression level by the same concentration of inducer differs between the laboratory and industrial scale, and in some cases, increasing the inducer concentration mediates significant toxicity, especially for the production of human therapeutic proteins^3–5,10–12^. Other promoter-dependent expression systems are induced by a metabolic change in the cell or required adding a specific nutrient that needs media replacement and is often practically difficult to establish at an industrial scale^13,14^. Furthermore, some expression systems could be induced by the glucose repression and the inoculation of lactose like studier medium^15–17^. In 2018, Anilionyte designed an endogenous promoter (PthrC) by RNA sequencing that autoinduced during the early exponential growth phase. However, the regularity capability of PthrC promoter was lower in different media, compared to T7 promoter. Moreover, the function of this promoter in the expression of different recombinant proteins has not yet been fully studied^18^. In 2016, a Self-Inducible Expression (SILEX) system was developed for *E.* *coli* host. The designed system was assessed by the expression of different protein models from human, plant, and bacterial origins. The SILEX system is composed of two pET vectors that concurrently transformed into *E.* *coli* strain. The first plasmid encodes human heat shock protein 70 (Hsp70), and the second one encodes the gene of interest. The autoinduction mechanism in the SILEX system is based on the leaky expression of heat shock protein 70 under T7 promoter at the early phase of bacterial growth. It was hypothesized that expressed Hsp70 interacts with host glyceraldehyde-3-phosphate dehydrogenase (GAPDH). GAPDH is the sixth enzyme in the glycolysis pathway and its interaction with Hsp70 shifts the glycolysis pathway to lactose pathway, and finally leads to protein expression. However, the exact mechanism has not been delineated yet. The self-induction phenomenon in the SILEX systems was also investigated in different *E.* *coli* strains including BL21 (DE3), BL21 Star (DE3), and BL21 (DE3) pLysS. In BL21 Star (DE3) strain, the engineered mutation causes more stability of the mRNA and ultimately more stability of the leaked protein (Hsp70), thus, increasing Hsp70 expression increases the autoinduction of target protein in this strain compared to other species. However, in *E.* *coli* BL21 (DE3) pLysS strain due to less leaky expression, the autoinduction mechanism is more inhibited^19^. The SILEX system drives protein expression at different temperatures, ranging from 20 to 37 °C, and culture media including Luria–Bertani broth (LB), Terrific broth (TB), and Yeast extract and tryptone media (2YT)^19^. The SILEX systems are supposed to be a simple cost–benefit replacement for other self-inducible systems that require specific media or nutrient for induction. The expression of different protein models using the developed SILEX system revealed that there is no difference in the point of induction among the studied proteins^19,20^. Despite the advantages, the designed system imposes an additional metabolic load to the host due to the expression of extra Hsp70 protein encoded by the first plasmid^18^. Previous studies have suggested the potential for self-inducing heat shock proteins including Hsp40, Hsp70, Hsp90, and Hsp110 in Lac-promoter-based expression systems^20^. Therefore, in the current study, for the first time, the ability of less molecular weight human heat shock proteins on the induction of enhanced green fluorescent protein (EGFP), romiplostim, and interleukin-2 was investigated. Moreover, the expression levels were also compared with an IPTG-induced expression system containing only the reporter gene plasmid. The plasmid stability was evaluated in three self-induced expression systems (Hsp70, Hsp40, and Hsp27) over 500 days. In addition, the expression stability was monitored using fluorescent signals for 90 days. The bacterial growth curves for SILEX and inducible systems were also investigated.

## Results

### Fluorescence signal quantification

**Figure 1:**
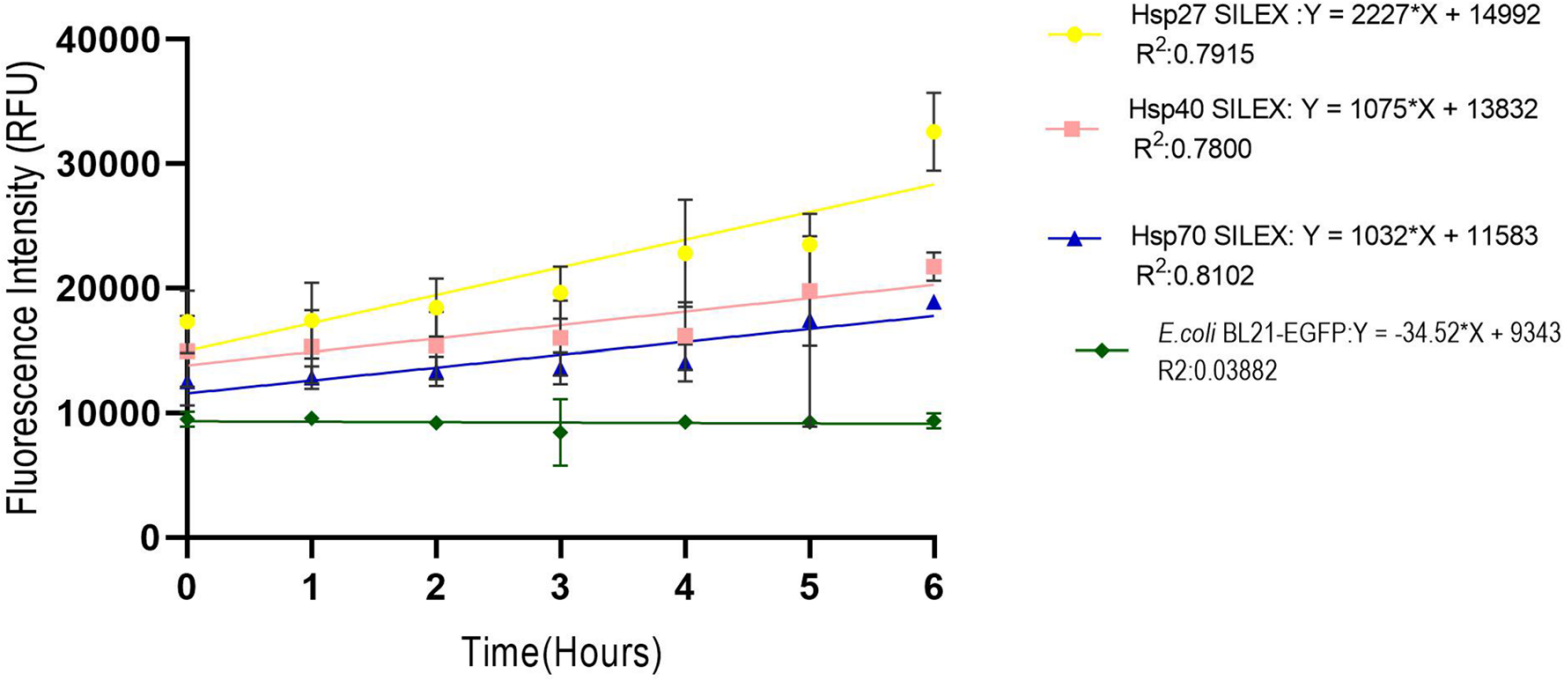
The intensity of fluorescence signals within 6 h incubation. The average of the fluorescence signal intensity for 10 different clones from SILEX systems was plotted for 6 h incubation. *Escherichia coli* Bl21 (DE3) containing plasmid pET28a-EGFP was evaluated without adding an inducing agent to observe the basal expression of the EGFP protein (Negative control). Data are represented as Mean ± SD from ten independents clones.

**Figure 2:**
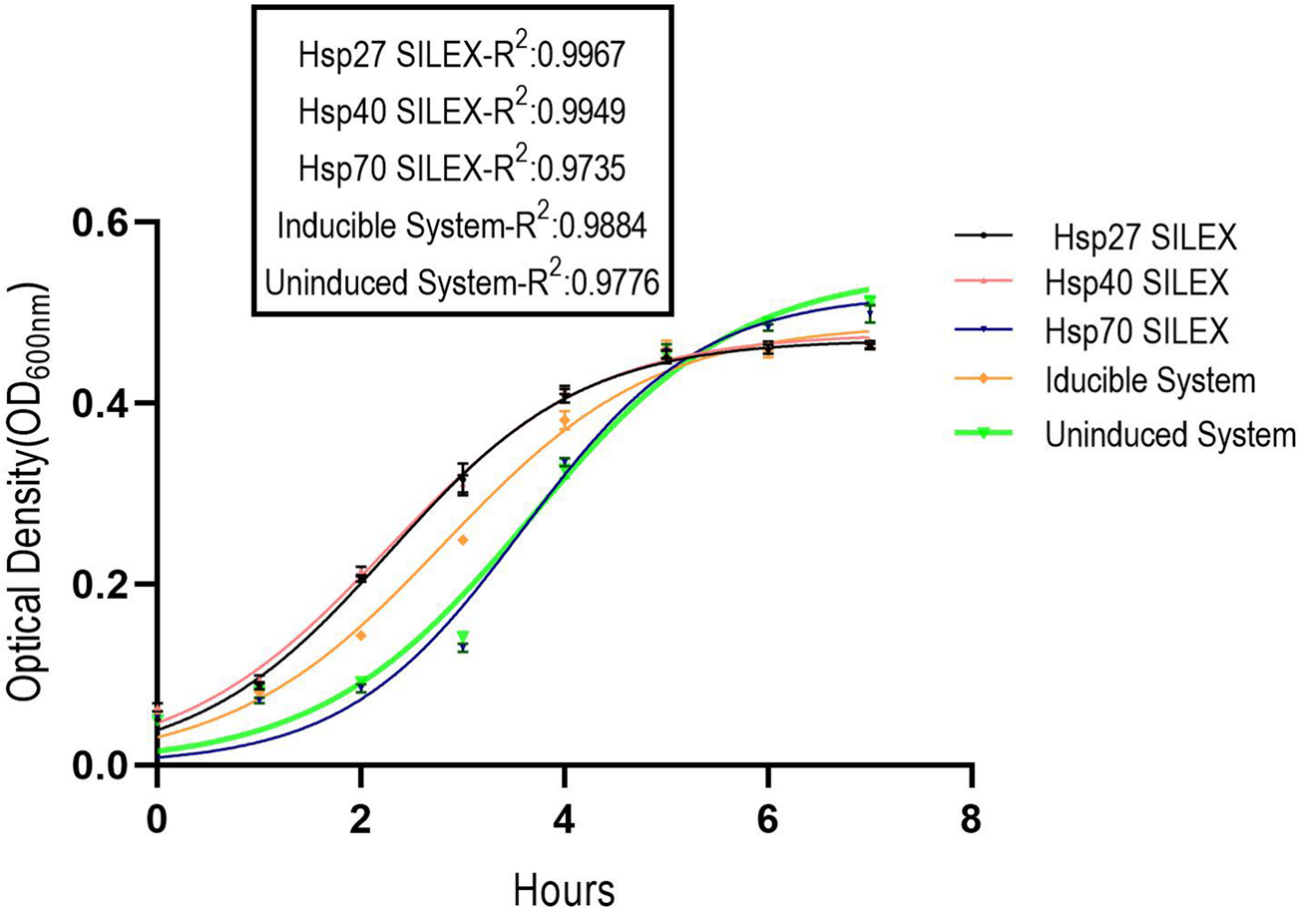
The nonlinear regression (Logistic growth) of bacterial growth curves for the inducible and autoinducible expression systems. *Escherichia* *coli* BL21 (DE3) strain containing pET28a-EGFP was evaluated as a control. Data are represented as Mean ± SD form three independent measurements.

To investigate the protein expression level during the time, the average of the fluorescence signal intensity for 10 different clones from SILEX systems and IPTG-inducible system were plotted for 6 h incubation at 37ºC/90 rpm. The fluorescent signal sensitivity was adjusted in the fluorimeter device because strong signals get out of the detection limit and weak signals interfere with background signal^21^. As depicted in Fig. 1, an increase in the fluorescent intensity was observed in all expression systems during 6 h incubation. Therefore, the comparison of protein expression between expression systems can be done in the shortest time of incubation at a micro-scale using fluorescent signals. As shown in Fig. 2, Hsp70 and inducible systems exhibit a more lag-time and faster growth rate than Hsp27 and Hsp40 SILEX systems. The slopes obtained by linear regression in the logarithmic phase were 0.09, 0.09, 0.11, 0.10, and 0.11 (RFU/h) for Hsp27, Hsp40, Hsp70, inducible, and uninducible system (as control), respectively (Supplementary file, Fig. S1). The maximum and minimum slopes in the logarithmic phase were obtained for the uninducible system, Hsp70 SILEX systems and Hsp27, Hsp40 SILEX systems, respectively. The relationship between the absorbance (OD_600nm_) and the bacterial cell numbers was used to compare the expression level of EGFP in both inducible and autoinducible expression systems. The result showed, there is a linear correlation between the absorbance (OD_600nm_) and the bacterial cell numbers in the expression systems (Supplementary file, Fig. S2). The cell count calibration curves were used for the estimation of EGFP expression per cell in both SILEX and inducible systems.

### Plasmid and expression stability

**Figure 3:**
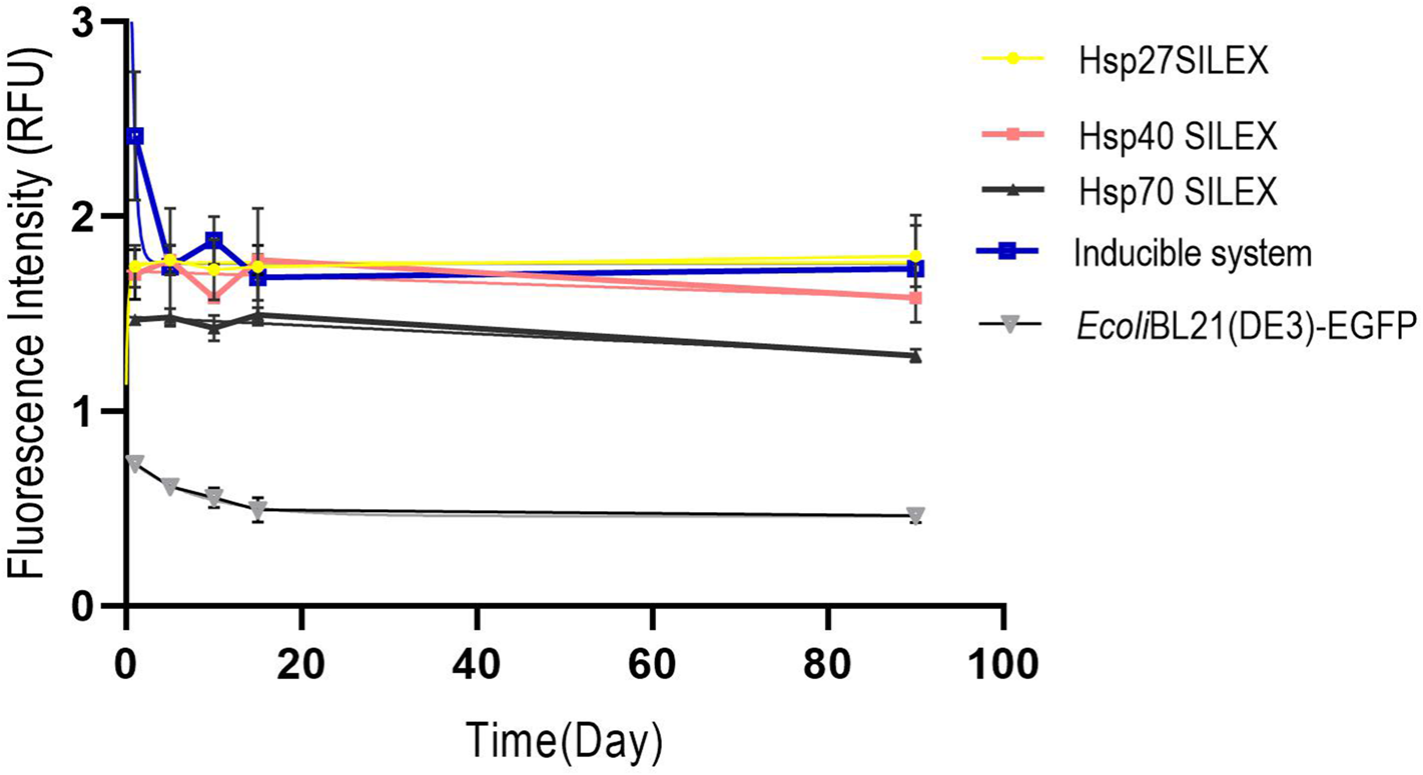
The expression level monitoring of transformed bacteria after four freeze-thawing cycles at − 70 °C within 90 days. *Escherichia* *coli* Bl21 (DE3) containing plasmid pET28a-EGFP was also evaluated without adding the inducing agent to observe the basal expression of the target protein. Data are represented as Mean ± SD from three independent measurements.

The plasmid stability of SILEX systems was investigated within 500 days with an interval of 10 days. The plasmid stability was confirmed by a PCR test using universal T7-Promoter and T7-Terminator primers (Supplementary file, Fig. S3). Moreover, the monitoring of EGFP expression levels during four cycles of freeze–thaw in SILEX systems (Fig. 3) showed that expression levels in Hsp27 were stable and more than Hsp70 and Hsp40 SILEX systems.

### Expression analysis using polyacrylamide gel

**Figure 4:**
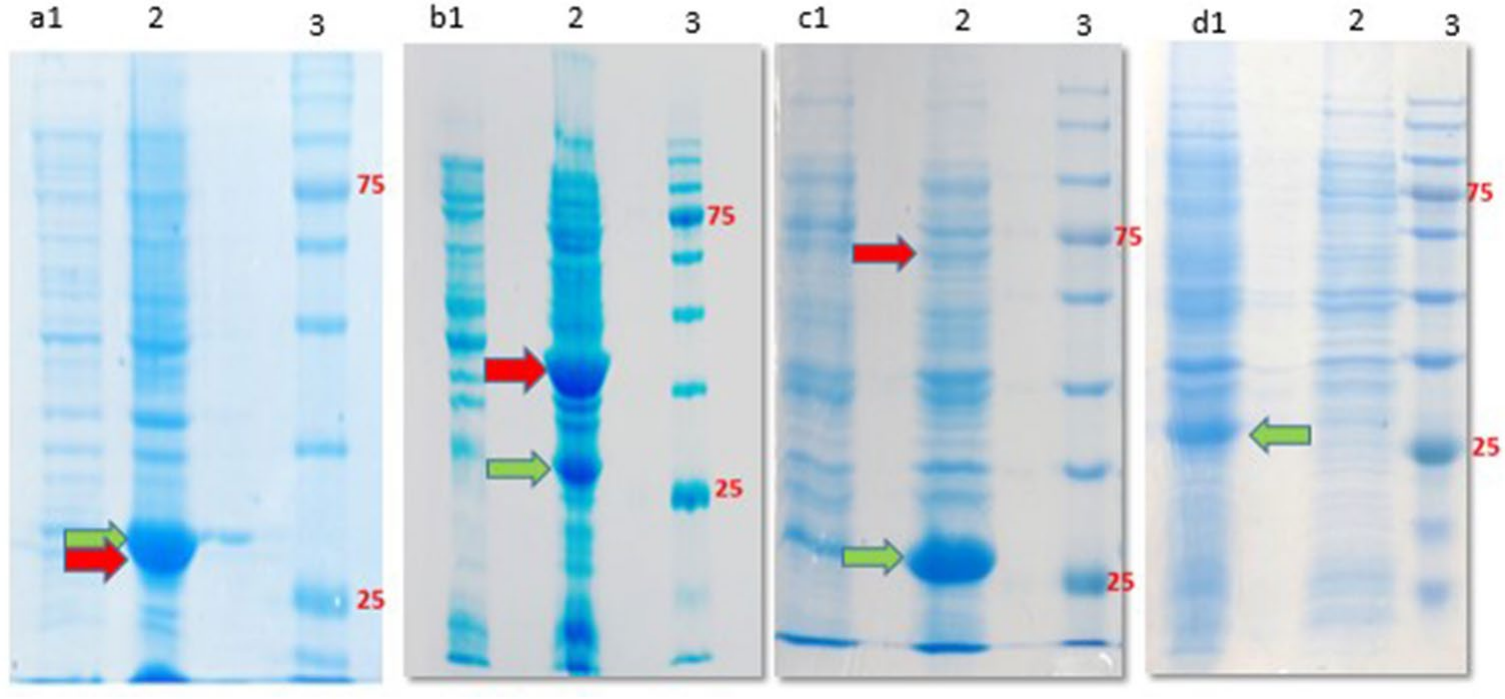
The SDS-PAGE analysis of EGFP expression in inducible and autoinducible systems. (**a**) Hsp27 SILEX system [lane 1: 2 h after inoculation, Lane 2: 16 h after inoculation, and lane 3: protein marker], (**b**) Hsp40 SILEX system [lane 1: 2 h after inoculation, Lane 2: 16 h after inoculation, and lane 3: protein marker (kDa)], (**c**) hsp70 SILEX system [lane 1: 2 h after inoculation, Lane 2: 16 h after inoculation, and lane 3: protein marker], and (**d**) Inducible system [lane 1: after induction, lane 2: before induction, and lane 3: protein marker]. The green arrow indicates the expression of EGFP and the red arrow indicates the leaky expression of the Hsp27, Hsp40, and Hsp70 on the gel a, b, and c, respectively. The protein marker also shows proteins with the molecular weights of 180, 135, 100, 75, 63, 48, 35, 25, 17, 11 kDa.

**Figure 5:**
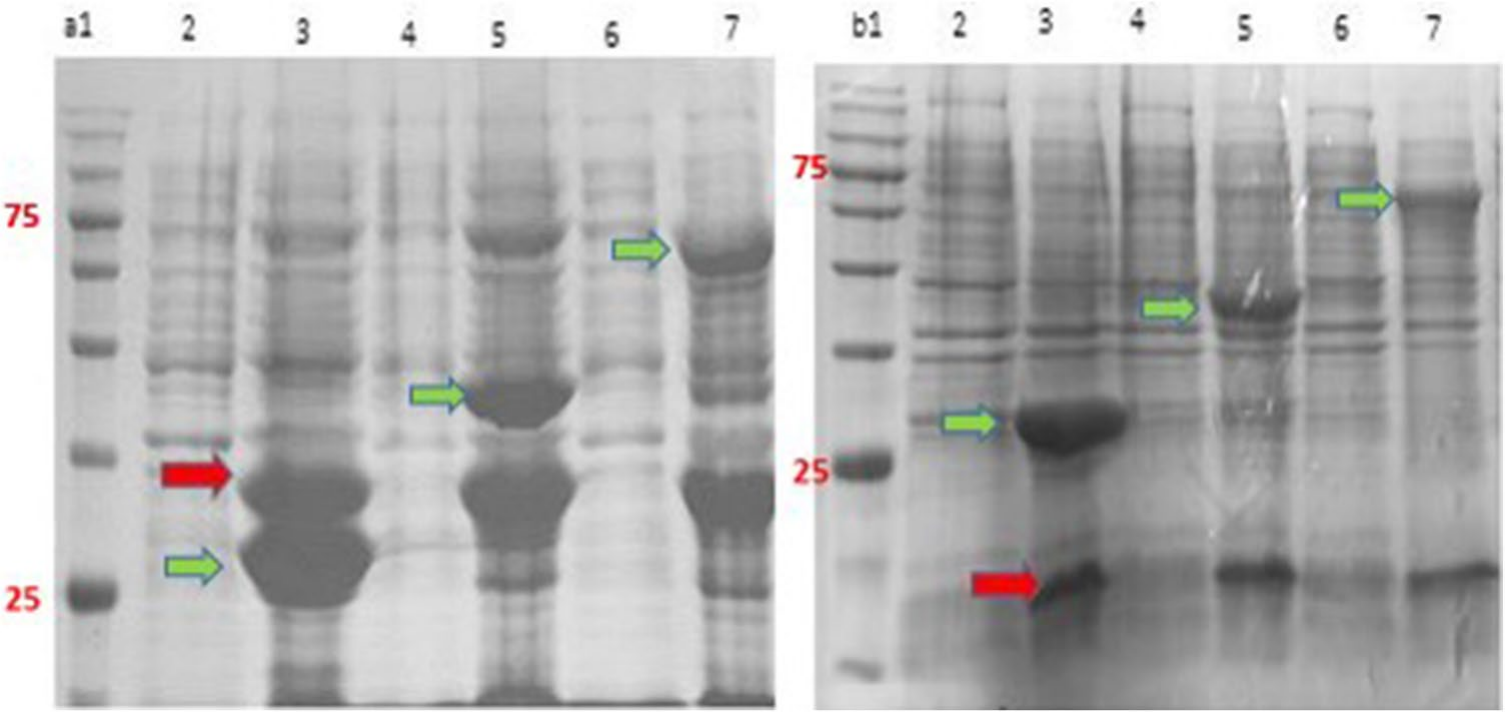
The SDS-PAGE analysis of expression of romiplostim and interleukin-2 in the SILEX systems. (**a**) Romiplostim: Hsp27 SILEX system [lane 2: 2 h after inoculation, Lane 3: 16 h after inoculation], Hsp40 SILEX system [lane 4: 2 h after inoculation, Lane 5: 16 h after inoculation], Hsp70 SILEX system [lane 6: 2 h after inoculation, Lane 7: 16 h after inoculation], and lane 1: protein marker. (**b**) Interleukin-2: Hsp27 SILEX system [lane 2: 2 h after inoculation, Lane 3: 16 h after inoculation], Hsp40 SILEX system [lane 4: 2 h after inoculation, Lane 5: 16 h after inoculation], Hsp70 SILEX system [lane 6: 2 h after inoculation, Lane 7: 16 h after inoculation], and lane 1: protein marker. The red arrow indicates the expression of romiplostim and interleukin-2 and the green arrow indicates the leaky expression of Hsp27, Hsp40, and Hsp70 on the gel a, b, and c, respectively. The protein marker also shows proteins with the molecular weights of 180, 135, 100, 75, 63, 48, 35, 25, 17, 11 kDa.

The EGFP expression was analyzed by 12% SDS-PAGE in the inducible and autoinducible systems. The stained gels showed that the protein was expressed in Hsp40 and Hsp27 SILEX systems similar to IPTG-inducible and Hsp70 SILEX systems with a sharp band at ~ 28 kDa (Fig. 4). The EGFP expression in three SILEX systems was confirmed using western blotting technique (Supplementary file, Fig. S4). In addition to the green fluorescent protein, romiplostim and interleukin-2 expressions were also assessed by 12% gel polyacrylamide in SILEX systems with sharp bands at ~ 30 kDa and ~ 16 kDa, respectively (Fig. 5). Figure S5 (Supplementary file) shows the expression of EGFP in the SILEX systems at different time points (0, 2, 8, and 16 h) after inoculation. As depicted in the figure, in all SILEX systems, the EGFP expression is time-dependent, in which at 8 h and 16 h of incubation the highest levels were obtained. Figure S6 (Supplementary file) shows the leaky expression of heat shock proteins (Hsp27, Hsp40, and Hsp70) at different time points (0, 2, 8, and 16 h) after incubation in the absence of EGFP plasmid. As exhibited in the figure, there is a basal expression of heat shock proteins with the same pattern in all SILEX systems. Figure S7 (Supplementary file) shows the IPTG-induced expression of heat shock proteins (Hsp27, Hsp40, and Hsp70) at 2 h and 16 h after inoculation in the absence of EGFP plasmid. As indicated in the figure, a sharp expression band was observed for heat shock proteins after 16 h IPTG-induction. Also, the expression of EGFP, interleukin-2, and romiplostim protein in 1-L cultures was confirmed by 12% SDS-PAGE for all SILEX systems (Supplementary file, Fig. S8, S9, and S10, respectively). Moreover, the purification of EGFP, interleukin-2, and romiplostim protein was performed using affinity chromatography for all SILEX system and analyzed by 12% SDS-PAGE (Supplementary file, Figs. S11, S12, and S13, respectively). The concentration of purified proteins was calculated by ImageJ software in three independent measurements. Among auto-inducible systems, Hsp27 SILEX system showed the highest protein yield for all tested proteins. The statistical analysis revealed that there is a significant difference in the concentration of purified proteins (EGFP, interleukin, and romiplostim) between Hsp27 system and two other SILEX systems (Table S1).

### Expression analysis using fluorimetry

**Figure 6:**
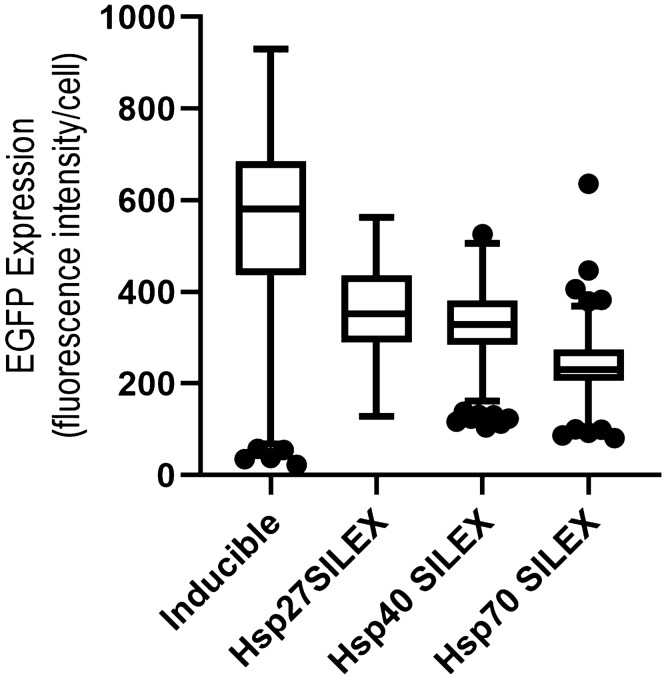
Box plot graph of the protein expression levels in the expression systems. As result depicted, a higher variation was observed for the IPTG-inducible system in contrast to the SILEX systems.

The Shapiro–Wilk normality test indicated that the EGFP expression levels do not normally distribute in the different clones of expression systems except for Hsp27 SILEX system (p-value = 0.193). Because the expression systems didn’t have a normal distribution of data, a non-parametric Kruskal–Wallis test was utilized to compare the expression levels between the groups. Among auto-inducible systems, Hsp27 SILEX system has the highest EGFP expression level. The statistical analysis revealed that there are significant differences in the expression level of EGFP between inducible and Hsp70 SILEX systems (p-value = 0.0000), inducible and Hsp40 SILEX systems (p-value = 0.0000), Hsp70 and Hsp40 SILEX systems (p-value = 0.006), Hsp70 and Hsp27SILEX systems (p-value = 0.000), and inducible and Hsp27 SILEX systems (p-value = 0.001). However, no significant difference was observed between Hsp40 and Hsp27 SILEX systems (p-value = 0.261). As summarized in Table 1, the minimum and maximum expression levels among different clones were for the IPTG-inducible system. Moreover**,** box plot analysis indicated that the highest variation was for the IPTG-inducible system when compared to the SILEX systems (Fig. 6).

**Table 1:** Descriptive analysis of EGFP Expression in SILEX and inducible systems.

Expression system	Minimum expression (RFU/ml)	Maximum expression (RFU/ml)	Range^a^	Median	Mean Rank^b^
Inducible	22.70	930.55	907.84	580.4888	143.38
Hsp27 SILEX	128.39	562.20	433.81	352.6144	105.58
Hsp40 SILEX	104.93	526.37	421.44	329.1599	92.58
Hsp70 SILEX	80.95	636.65	555.40	230.6803	60.46

^a^The difference between the lowest and highest values in each expression system.

^b^Ranking all the values from low to high, and then compared the means ranks.

## Discussion

Recently, a new autoinducible system was proposed that uses a lac inducible plasmid containing Hsp70 sequence. This expression system does not require any special media or inducer for protein expression and works well in a diverse and common culture media (e.g., TB, LB, M9, 2YT, and YTA)^19^. Moreover, it is not required optical density monitoring for induction at an optimal point and has been suggested to be used in a wide range of temperatures (ranging from 20 to 37 °C). In addition, like other expression systems, affinity chromatography can be used as a fast and simple method for these systems based on the interaction between the protein and a specific ligand. The design of suitable tags for the protein of interest facilitates the purification process. For example, in the current study, to isolate the protein, a c-terminus His-tag was used for purification of the target protein using IMAC system^19,20^.

Previous studies showed that coexpression of recombinant proteins and chaperones improves the expression level of recombinant proteins in *E.* *coli* host^22^. In this regard, heat shock proteins including DnaK (human Hsp70) and DnaJ (human Hsp40) are the most prominent chaperones^23,24^. Hsp70 protein mediates accurate assembly and folding of newly synthesized polypeptide segments and prevents protein aggregation in response to stress^25–27^. Briand *et.al* proposed that Hsp70 interacts with GAPDH enzyme and this interaction shifts the glycolysis pathway to lactose pathway and finally leads to protein expression. However, the exact mechanism of Hsp70 SILEX system has not been delineated yet and further studies should be done^20^. The expression of Hsp70 imposes an extra metabolic load on the host as revealed by our results that the expression level in Hsp70 SILEX system is lower than the other two SILEX systems. Hsp40 is another highly conserved group of molecular chaperons that overexpresses in response to stress factors. It binds to different substrates like proteins and peptides and delivers them to Hsp70. Simultaneously, it modulates the ATPase activity of Hsp70 to refold the substrates^25,28,29^. Hsp27, also known as human HspB1, is an ATP-independent small protein that similarly protects cellular misfolded proteins from aggregation inside the cells and refolds them with the help of other ATP-dependent chaperones^30,31^. A study by Wang et al*.* revealed that overexpression of Hsp70 in the cell leads to more glucose consumption and higher glycolytic enzyme activities without a significant change in the ATP level due to decreased activity of oxidative phosphorylation^32^. In short, glycolysis pathway was more used for the compensation of ATP balance. By depletion of the glucose, the lactose pathway is activated that leads to the protein expression. Although Wang et al*.* and Briand *et.al* studies are controversial, both of them can somehow explain the induction of protein expression. Also, other studies have shown that under heat and stress conditions, the cells increase the expression of heat shock proteins and use more glycolysis pathway to produce the energy needed for proper folding and prevention of degradation and denaturation of proteins^33^. This hypothesis could bring up for Hsp27 and Hsp40 SILEX systems, in which the glucose consumption drives the cells to use other carbon sources like lactose that leads to the expression of recombinant protein^34^. This hypothesis is in good agreement with the functional mechanism of self-inducing media such as ZYM5052 and MDA5052^16,32,35,36^. In these self-induced media, the expression systems preferentially uptake the existence glucose of the medium as a carbon source. In glucose depletion condition, lactose is used as an alternative carbon source. It is converted to an allolactose by beta-galactosidase that acts as an inducing agent leading to spontaneous expression of the target proteins by binding to the repressor^37^. Therefore, in the current study, for the first time, the ability of Hsp27 and Hsp40 on the induction of EGFP expression was investigated. The SDS-PAGE analysis indicated that Hsp27 and Hsp40 proteins could induce the EGFP expression in the bacterial cells similar to Hsp70. The fluorimetric results elucidated that the level of protein expression in the Hsp27 and Hsp40 systems is significantly higher than Hsp70. Nonetheless, the highest expression level was found to be for the inducible system.

The Hsp70 SILEX and inducible systems showed a more lag-time in the bacterial growth curve than Hsp27 and Hsp40 systems. The lag-phase is time to adapt the bacteria to a new enriched condition. The duration of lag-phase depends on the bacterial species, synthesis of necessary components for bacterial growth, macromolecular damage induced by previous stationary stage, inoculum size, the heterogeneity rate of inoculum in bacterial mass, plasmid size, and physicochemical properties of the previous and the new culture media^38–40^. Since the size of the inoculum is assumed to equal for all SILEX systems, the longer lag-phase in the Hsp70 SILEX system could be attributed to a higher metabolic burden of Hsp70 plasmid on the host or even unknown molecular and physiological mechanisms in the bacterial cells. Besides, since the lag-phase involves the synthesis of proteins and crucial cellular components for the bacterial growth and as well as the repair of molecular damage, the expression of heat shock proteins can have a supporting role in such cellular processes that in turn, reduce the duration of lag-phase in autoinducible systems compared to the inducible system. On the other hand, Hsp27 and Hsp40 SILEX systems showed a lower growth rate (slopes in the logarithmic phase) in the bacterial growth curves than Hsp70 and inducible systems. Since the logarithmic phase is a stage of bacterial growth that the cells divide exponentially by binary fission, any induction of recombinant protein expression at this stage significantly affects the rate of cell division and bacterial growth^41^. Thus, in Hsp27 and Hsp40 SILEX systems, because the bacteria have a higher expression of recombinant proteins, the slope of logarithmic phase is lower than other expression systems (Hsp70 and inducible systems).

There is more variation in the EGFP expression levels between different transformed colonies in the inducible system than SILEX systems. This phenomenon can be explained by this fact that heat shock proteins constantly self-metabolized by transformed bacteria and continuously self-induced the expression of target protein in the host during incubation time^37^, whereas, synthetic inducers like IPTG need to pass through the bacterial cell membrane via LacY-mediated active transport. Therefore, due to the cell-to-cell differentiation and phenotypic heterogeneity, different concentrations of the inducer are received by the bacterial cells, which lead to a high variation in the protein expression between cells in the inducible system. Moreover, the high expression variability in the inducible system can also be described by leaky expression, plasmid loss, a mutation on the plasmid, and chromosomal mutations that lead to inappropriate function of T7 RNA polymerase^12,42^.

Considerably, auto-inducible systems showed a stable EGFP expression after multiple freeze–thaw cycles during 90 days, which may be attributed to the presence of heat shock proteins. Despite small variations in the expression level, no downward trend was observed for SILEX systems. This is because the expression of heat shock proteins naturally increases in response to temperature stress, the presence of an excessive antibiotic or toxin, hypoxic conditions, and extreme pH. Therefore, the simultaneous expression of heat shock proteins with the target protein can rapidly prevent the instability and the stress induced by freeze–thaw cycles^43^. This is more evident in the inducible system, in which a remarkable decrease in the expression was observed after the first freeze–thaw cycle without further decline until 90 days. The formation of ice crystals at low temperatures and dehydration affects the expression of proteins in the bacteria cells at the early stages. However, these changes, in turn, trigger the expression of heat shock proteins that finally leads to the resistance of bacteria to the repeated freeze–thaw cycles^44–46^.

## Conclusion

In conclusion, Hsp27 could be suggested as a suitable autoinducer to induce target protein in Lac promoter-based *E. coli* expression systems because of less variability in the expression among different clones, less metabolic load on the host, good expression stability after several freeze-thawing cycles, and most importantly, a higher mean rank of protein expression than other SILEX systems.

## Methods and materials

### Bacterial strains, plasmids, and growth condition

*Escherichia coli* DH5-ɑ and *E.* *coli* BL21 (DE3) strains were used for amplifying the plasmids and expression of proteins, respectively. All strains were obtained from the national cell bank of Iran (Pasteur Institute of Iran). The *hsp27* (P04792), *hsp40* (P25685), *hsp70* (P0MDV8), and *egfp* genes (pEGFP plasmid, Addgene, USA) were synthesized by Biomatic company (Canada). The *egfp* gene was cloned into pET28a using *NcoI* and *XhoI* restriction sites. The *hsp70*, *hsp40*, and *hsp27* genes were cloned into pET21a plasmid using *NdeI* and *XhoI* restriction sites to construct pET21-Hsp70, pET21a-Hsp40, and pET21a-Hsp27, respectively. Plasmids were transformed into *E.* *coli* BL21 (DE3) via the heat-shock method^47^. Bacterial cells were cultured in Luria Bertani broth (LB) medium at 37 ºC supplemented with an antibiotic (50 µg/ml kanamycin or 100 µg/ml ampicillin) depend on the recombinant plasmids.

### Fluorescence signal quantification

The fluorescence intensity was measured for 10 different clones of SILEX systems (inoculation of 190 µl LB broth with 10 µl overnight bacterial suspension) within 6 h incubation at 37ºC/90 rpm at 1 h intervals. The experiment was repeated three times for each clone. The fluorescence signals were measured at an excitation of 485 nm and emission of 528 nm (fluorimeter, BioTek, USA). Also, the basal expression of target protein was evaluated by monitoring of fluorescence intensity in the colonies containing pET28a-EGFP for 6 h without addition the inducing agent.

### Bacterial growth curves

The optical density of each expression system (*E.* *coli* transformed with either pET28a-EGFP and pET21a-Hsp70 or pET28a-EGFP and pET21a–Hsp40 or pET28a-EGFP and pET21a–Hsp27) was measured using a microplate reader (Epoch, BioTek, USA) at a wavelength of 600 nm. Briefly, 10 µl of overnight cultures were inoculated into a 96 well-microplate containing 190 µl LB Broth supplemented with the appropriate antibiotics and incubated at 37ºC/90 rpm and the optical densities (OD_600nm_) were monitored for 7 h at 1.5 h intervals in triplicate. The data was used for plotting of bacterial growth curve. This curve was also plotted for *E.* *coli* BL21 (DE3) transformed with pET28a-EGFP in induced and uninduced conditions.

### Bacterial cell counting

A single clone of each expression system (Hsp70, Hsp40, and Hsp27 SILEX and inducible systems) was inoculated into 5 ml LB medium supplemented with the appropriate antibiotics and incubated for 16 h at 37 ºC/170 rpm. The cultures were then diluted 50-fold in LB broth. Then 200 µl of each diluted culture was added to a 96-well microplate and incubated at 37 ºC, 90 rpm. The optical density (OD_600nm_) was then monitored at 1.5 h intervals for 7 h for cell counting. At the same intervals, 10 µl of each bacterial culture was removed and diluted 10@@@4 times by phosphate buffer saline (PBS). Then, 5 μl of the diluted sample was spread on LB agar containing 35 μg/ml kanamycin and 100 µg/ml ampicillin. The clones were counted in triplicate after 24 h incubation at 37 ºC. The bacterial count plot was obtained from the optical density versus counted clones.

### EGFP expression in SILEX and IPTG-inducible systems

Fifty clones containing either pET21a-Hsp70 or pET21a-Hsp40 or pET21a-Hsp27, and pET28a-EGFP plasmids were separately inoculated into 50 tubes containing 5 ml LB medium, 35 μg/ml kanamycin, and 100 µg/ml ampicillin and allowed to grow at 37ºC, 170 rpm overnight. Then, 10 µl bacterial culture of each tube (OD600nm ~ 2.5) were transferred into a 96- well microplate containing 190 µl LB Broth in triplicate and incubated at 37ºC,90 rpm for 6 h. The protein expression in each colony was then measured using fluorimetry (485 nm excitation and 528 nm emission) and “OD_600nm_ vs. cell count” standard curve. Concurrently, 50 clones containing pET28a-EGFP plasmid were inoculated into 50 tubes having 5 ml LB medium supplemented with 50 μg/ml kanamycin and allowed to grow at 37ºC/170 rpm overnight. Then, 10 µl cell suspension of each clone (OD_600nm_ ~ 2.5) were inoculated in triplicate into a 96 well-microplate containing 190 µl LB Broth supplemented with 50 μg/ml kanamycin and incubated at 37ºC/90 rpm. The expression was induced by adding IPTG with a final concentration of 1 mM at OD_600nm_ of 0.6. The EGFP expression in each colony was finally measured 6 h after inoculation using fluorimetry (485 nm excitation and 528 nm emission) and “OD_600nm_ vs. cell count” standard curve.

### Plasmid stability

Randomly, 20 different clones from each SILEX system were cultured on LB agar plates containing 35 µg/ml kanamycin and 100 µg/ml ampicillin. The clones were then subcultured every 10 days on agar plates. The stability of plasmids was confirmed by PCR test after 500 days of subculturing using T7-promoter and T7-terminator primers.

### EGFP expression stability

EGFP expression in each expression system was monitored in triplicate after several freeze-thawing cycles at -70 °C. Briefly, the bacteria were transformed with the appropriate vectors. Then, the EGFP expression level in each transformed clone was measured using fluorimetry. The glycerol stocks were then prepared and stored at -70 °C for 5 days. After the thawing step, 100 µl of glycerol stocks were inoculated into 5 ml of LB medium supplemented with selective antibiotic and incubated in a shaker incubator at 37ºC/170 rpm, overnight. Then, 100 µl of seed cultures was added to 5 ml of LB medium and auto-induced for an overnight at 37ºC/250 rpm. In the inducible system, the expression was induced by adding 1 mM IPTG at OD_600nm_ ~ 0.6 for an overnight. The EGFP expression level in each expression system was again measured using fluorimetry. This freeze-thawing cycle was repeated four times for all expression systems and EGFP expression stability was monitored for 90 days.

### Expression analysis using SDS-PAGE and western blot

In addition to fluorimetry assay, the EGFP, romiplostim, and interleukin-2 expressions were assayed by SDS-PAGE at different time points. Randomly, a single clone of each autoinducible SILEX and inducible expression system was inoculated into 5 ml of LB medium and incubated at 37ºC, 170 rpm for 18 h. Then, 100 µl of bacterial culture was added to 5 ml LB medium and incubated for another 18 h at 37ºC, 250 rpm. The induction of inducible system was performed by the addition of 1 mM IPTG at OD_600nm_ ~ 0.6. The EGFP expression in SILEX and inducible systems was finally analyzed by 12% sodium dodecyl sulfate (SDS)-polyacrylamide gel electrophoresis and Coomassie blue staining method^48^. Moreover, the leaky and IPTG-induced expression of heat shock proteins were investigated in the absence of EGFP plasmid at different time points. Also, the EGFP expression in SILEX systems was assessed using western blot technique. Briefly, the proteins were blotted on nitrocellulose membrane and blocked with a TBST buffer (Tris-buffered saline, 0.1% Tween 20) containing 3% bovine serum albumin for 1 h. The blocked membrane was incubated with an anti-his antibody at a dilution of 1:2000 for 16 h at 4 °C. The membrane was then washed three times with TBST buffer and the protein bands were finally visualized by a PBS solution (50 ml) containing 30 mg DAB (3,3′-Diaminobenzidine) and 50 µl H_2_O_2_ 30%.

### Expression and purification in 1-L culture

The expression of EGFP, romiplostim, and interleukin-2 in 1-L culturing flasks was assayed for all SILEX systems. Randomly, a single clone of each system was inoculated into 30 ml LB medium and incubated at 37ºC, 170 rpm for 18 h. Then, 25 ml bacterial culture was added to 1 L LB medium supplemented with the selective marker and incubated for 18 h at 37ºC, 220 rpm. The expression in SILEX systems was finally analyzed by 12% sodium dodecyl sulfate (SDS)-polyacrylamide gel electrophoresis and Coomassie blue staining method. After the expression, the purification of EGFP and interleukin-2 was also performed for all SILEX systems using Ni–NTA column chromatography. In brief, the bacterial cells expressing the His-tagged proteins were lysed by 10 ml binding buffer (NaH2PO4.2H2O 50 mM, NaCl 300 mM, adjusted to pH 8) containing 1 mg/ml lysozyme and sonicated for 1 h on ice. The lysates were then centrifuged at 13,000 rpm for 25 min at 4 °C. The supernatants containing the His-tagged proteins were loaded on equilibrated Ni–NTA resins and stirred for 1 h at 4 °C. Then, the resin was washed with 10 ml wash buffer (binding buffer plus 20 mM imidazole) for three times. After that, the resin was washed with 3 ml elution buffer (binding buffer plus 250 mM imidazole) in 200 µl fractions. Moreover, the purification of romiplostim was performed using protein A affinity chromatography for all SILEX systems according to the previous study. In brief, the culture was centrifuged and the pellet was lysed by resuspension in a lysis solution (50 mM Tris HCl, 10% Sucrose, 1 mg/ml lysozyme, adjusted to pH 8) and subsequent sonication on ice (20 cycle, 20 s on, 20 s off). The lysate was then centrifuged at 5000 rpm/4 °C for 25 min. The pellet was resuspended in wash buffer (100 mM Tris HCl, 2 M Urea, 2% (w/v) Triton X100 and 5 mM EDTA, adjusted to pH 7) and centrifuged at 5000 rpm/4 °C for 25 min. This process was repeated three times and the resulted pellet was solubilized by a buffer containing 8 mM DTT, 8 M Urea, and 50 mM Tris (pH 10) for 1 h in a shaker incubator. Finally, the romiplostim protein was purified by passing to a protein A-Sepharose resin (Merck, USA) and elution by 20 mM sodium acetate buffer (pH 4)^49^.

### Statistical analysis and curve plotting

The data of the EGFP expression in SILEX and inducible systems were statistically analyzed using SPSS version 25. The normality of data was tested by Shapiro–Wilk's normality test. The difference between the expression systems was measured using Kruskal Wallis and post hoc tests. A p-value of less than 0.05 was considered as a significant difference between the groups. The curve fitting and regressions were performed using Graph Pad Prism 8.0.2. The logistic growth nonlinear regression model was used to fit the bacterial growth curves. Also, the concentration of expressed recombinant proteins was calculated by ImageJ software (https://imagej.nih.gov/ij/download.html) and the expression yields between SILEX systems were statistically compared using two way ANOVA test in Graph Pad Prism 8.0.2.

## Supplementary Information

Supplementary Information

## Data Availability

All data generated or analyzed during this study are included in the published article and supplementary file and or are available from the corresponding author on reasonable request.
