# Supplementary material for: Comparison of E. coli based self-inducible expression systems containing different human heat shock proteins
Source: Sci Rep. 2021 Feb 25;11:4576. doi: 10.1038/s41598-021-84188-8 (PMC7907268; doi:10.1038/s41598-021-84188-8)
Supplement: Supplementary file 1 — Supplementary Information [file 41598_2021_84188_MOESM1_ESM.pdf]

Supplementary data

Comparison of *E.coli* based self-inducible expression systems containing different human heat shock proteins

Fatemeh Sadat Shariati[1], Malihe Keramati[1], Vahideh Valizadeh[1], Reza Ahangari Cohan[*1], Dariush Norouzian[*1]

[1]Department of Nanobiotechnology, New Technologies Research Group, Pasteur Institute of Iran, Tehran, Iran

***Corresponding authors:**
Department of Nanobiotechnology, New Technologies Research Group, Pasteur Institute of Iran, Tehran, Iran, Fax: (98)2166465132, Tel: (98)2164112171, Emails: cohan_r@pasteur.ac.ir and dnsa@pasteur.ac.ir

(**a**) IPTG-inducible system

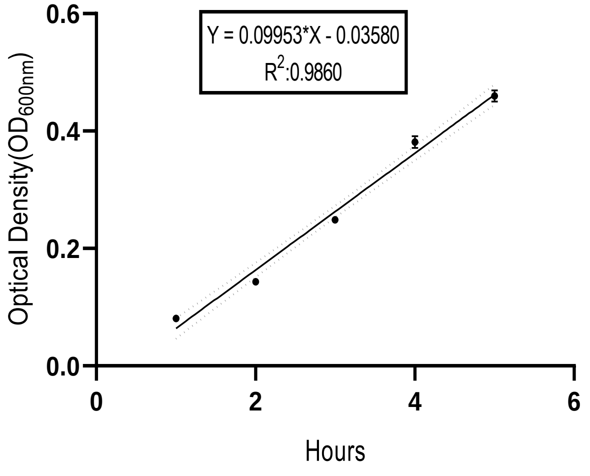

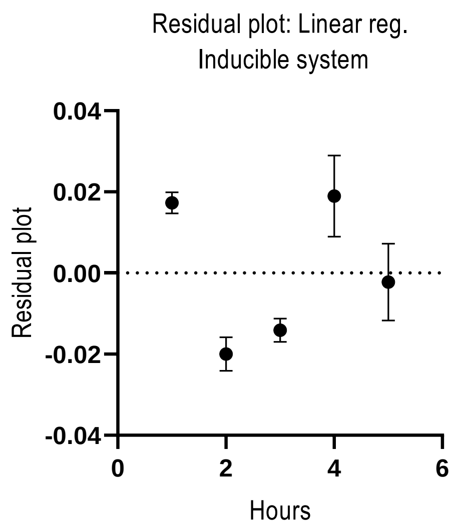

(**b**) Hsp27 SILEX system

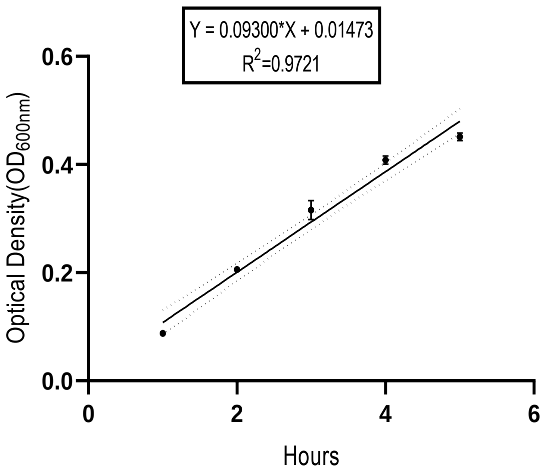

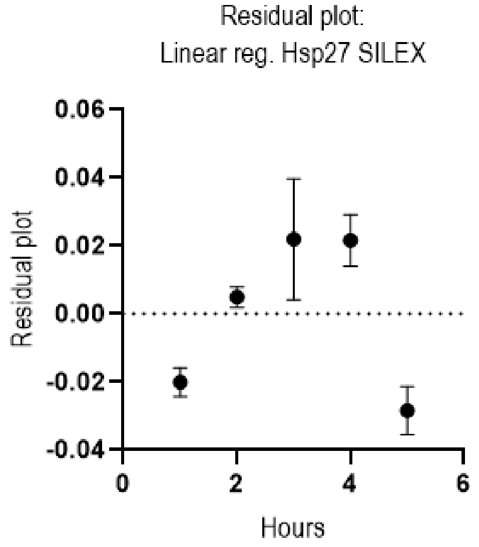

(**c**) Hsp40 SILEX system

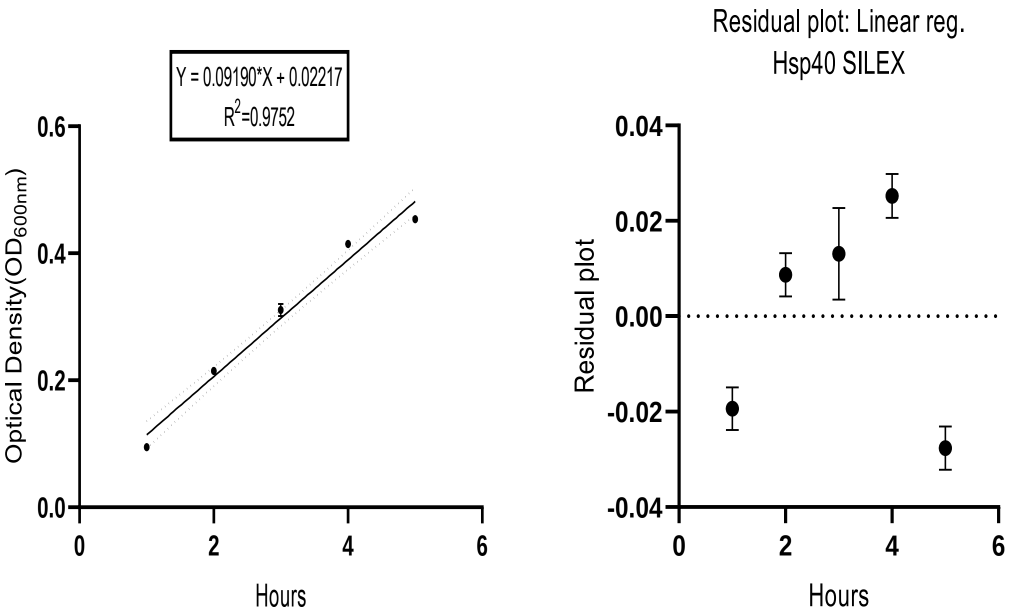

(**d**) Hsp70 SILEX system

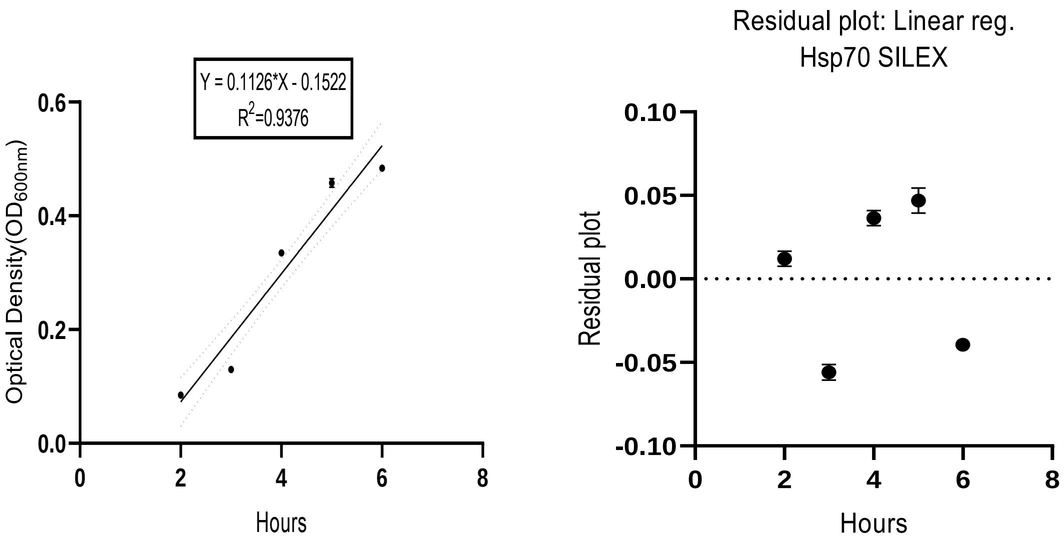

(**e**) Uninduced system

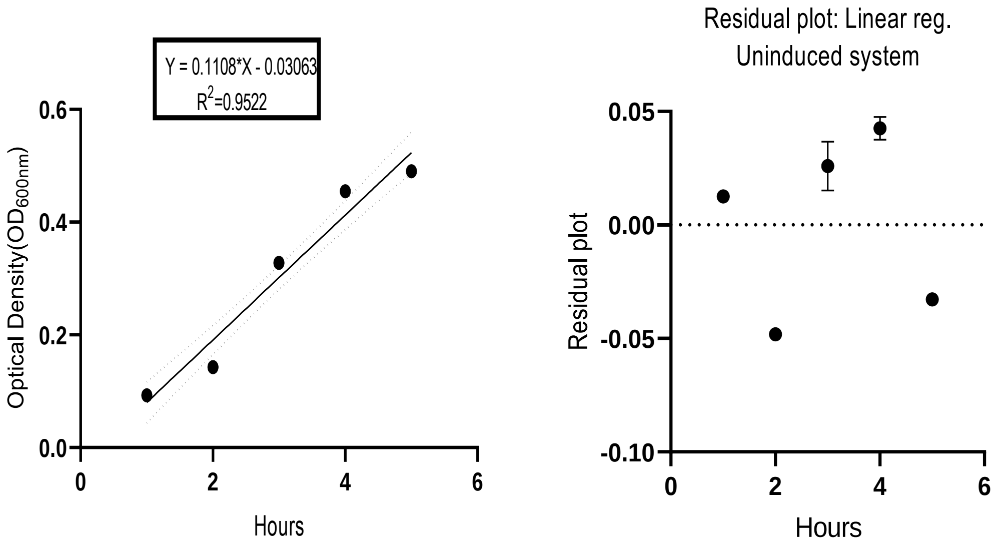

Figure S1. The linear regressions of bacterial growth curves at logarithmic phase. (**a**) IPTG-inducible system, (**b**) Hsp27 SILEX system, (**c**) Hsp40 SILEX system, (**d**) Hsp70 SILEX system, and (**e**) uninduced system (*E.coli* BL21 (DE3) strain containing pET28a-EGFP). Data are represented as Mean ± SD form three intendent measurements with a confidence interval of 95%.

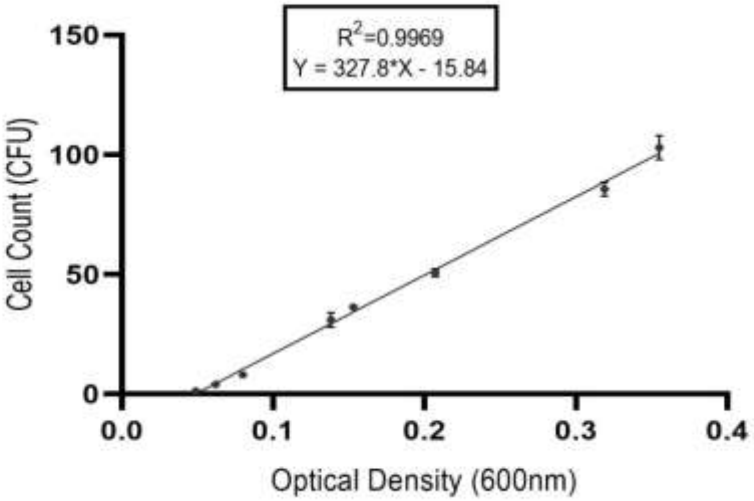

Figure S2. Cell counting curve. The cell density (OD_{600nm}) was plotted against the number of bacterial cells (CFU). Data are represented as Mean ± SD form three independent measurements.

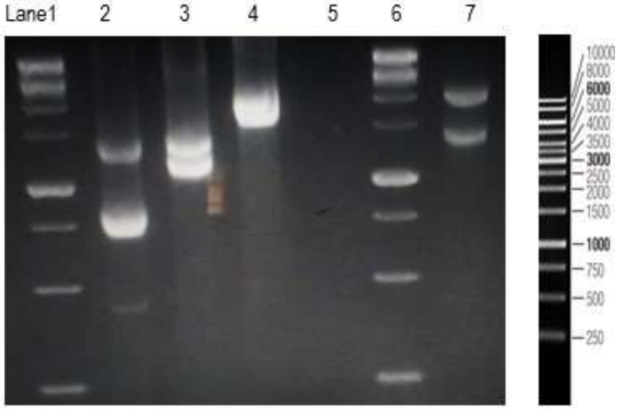

Figure S3. Colony PCR on double-transformed bacteria after 500 days of subculturing on 2% gel agarose. [Lane1 and 6 ; DNA Ladder, Lane 2; Hsp27 SILEX system containing pET21a-*hsp27* (619 bp) and pET28a-*egfp* (1024 bp), Lane 3; Hsp40 SILEX system containing pET21a-*hsp40* (1305 bp) and pET28a-*egfp* (1024 bp), Lane 4; *E.coli* BL21(DE3) containing pET21a-*hsp70* (1927 bp) as control, Lane 5; *E.coli* BL21(DE3) without plasmid, Lane7; Hsp70 SILEX system containing pET21a-*hsp70* (1927 bp) and pET28a-*egfp* (1024 bp)].

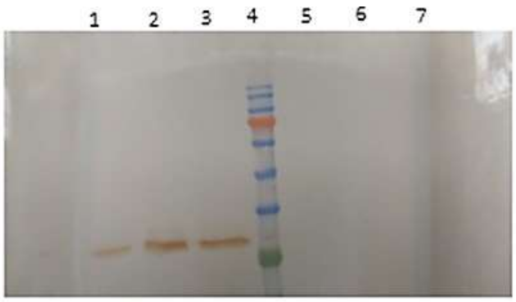

Figure S4. The western blot analysis of EGFP expression in autoinducible systems. [Lanes 1, 2, and 3: 16 h after inoculation for Hsp70, Hsp40, and Hsp27 SILEX system, respectively, Lane 4: protein marker, Lanes 5, 6, and 7: 2 h after inoculation for Hsp70, Hsp40, and Hsp27 SILEX system, respectively]. The protein marker also shows proteins with the molecular weights of 180, 135, 100, 75, 63, 48, 35, 25, 17, 11 kDa.

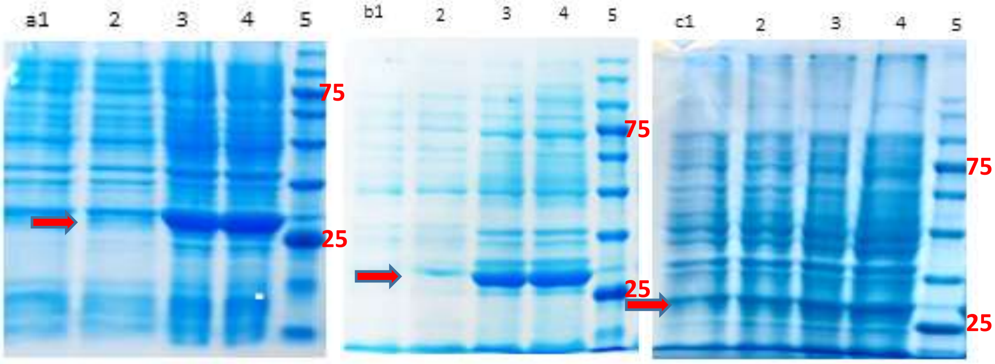

Figure S5. The EGFP expression in the SILEX systems at different time points. (**a**) Hsp27 SILEX system [lanes 1, 2, 3, and 4: 0, 2, 8, and 16 h after inoculation, respectively, and lane 5: protein marker], (**b**) Hsp40 SILEX system [lanes 1, 2, 3, and 4: 0, 2, 8, and 16 h after inoculation, respectively, and lane 5: protein marker], (**c**) Hsp70 SILEX system [lanes 1, 2, 3, and 4: 0, 2, 8, and 16 h after inoculation, respectively, and lane 5: protein marker]. The red arrow indicates the expression of EGFP on the gel. The protein marker also shows proteins with the molecular weights of 180, 135, 100, 75, 63, 48, 35, 25, 17, 11 kDa.

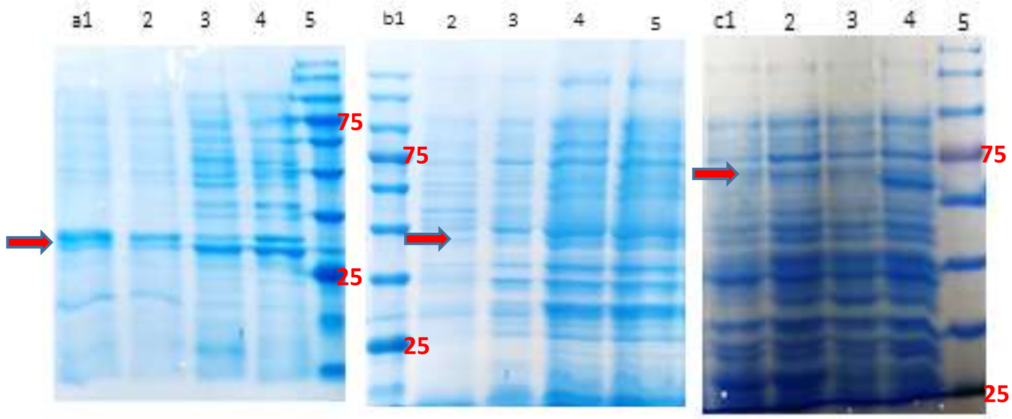

Figure S6. The SDS-PAGE analysis of heat shock proteins (Hsp27, Hsp40, and Hsp70) leaky expression at different time points in the absence of EGFP plasmid. (**a**) Hsp27 leaky expression [lanes 1, 2, 3, and 4 are 0, 2, 8, and 16 h after inoculation, respectively, and lane 5 is protein marker], (**b**) Hsp40 leaky expression [lane 1 is protein marker, and lanes 2, 3, 4, and 5 are 0, 2, 8, and 16 h after inoculation, respectively], (**c**) Hsp70 leaky expression [lane 1, 2, 3, and 4 are 0, 2, 8, and 16 h after inoculation, respectively, and lane 5 is protein marker]. The red arrow indicates the leaky expression of Hsp27, Hsp40, and Hsp70 on the gel a, b, and c, respectively. The protein marker also shows proteins with the molecular weights of 180, 135, 100, 75, 63, 48, 35, 25, 17, 11 kDa.

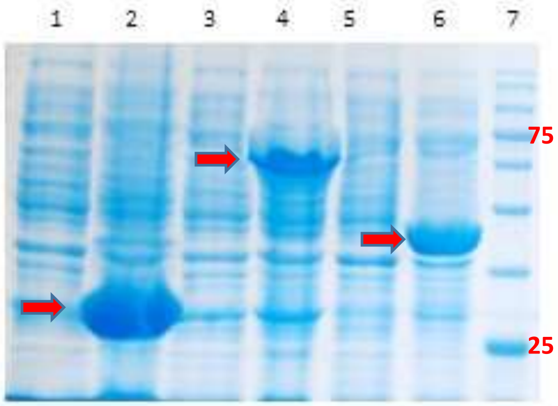

Figure S7. The SDS-PAGE analysis of Hsp27, Hsp40, and Hsp70 expression after IPTG induction at different time points in the absence of EGFP plasmid. [Lane 1: Hsp27 expression 2h after inoculation, Lane 2: Hsp27 expression 16h after inoculation, lane 3: Hsp70 expression 2h after inoculation, Lane 4: Hsp70 expression 16h after inoculation, lane 5: Hsp40 expression 2h after inoculation, lane 6: Hsp40 expression 16h after inoculation, and lane 7: protein marker]. The red arrow indicates the expression of Hsp27, Hsp70, and Hsp40 on the gel. The protein marker also shows proteins with the molecular weights of 180, 135, 100, 75, 63, 48, 35, 25, 17, 11 kDa.

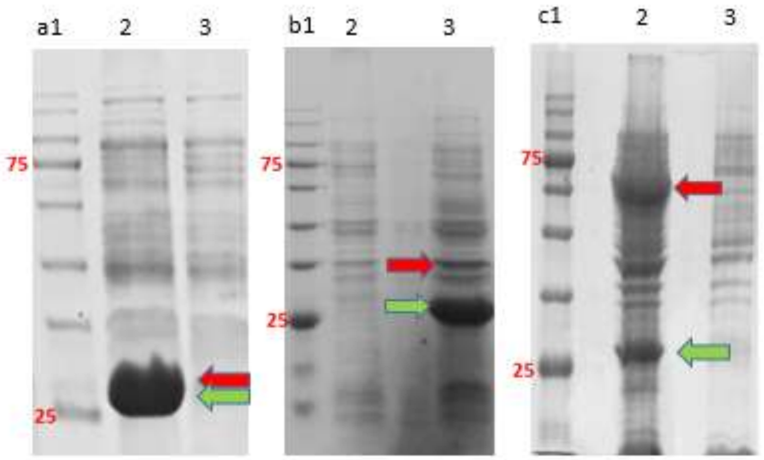

Figure S8. The SDS-PAGE analysis of EGFP expression in autoinducible systems. (**a**) Hsp27 SILEX system [lane 1: protein marker, lane 2: 16 h after inoculation, and Lane 3: 2 h after inoculation], (**b**) Hsp40 SILEX system [lane 1: protein marker, lane 2: 2 h after inoculation, and Lane 3: 16 h after inoculation], (**c**) hsp70 SILEX system [lane 1: protein marker, lane 2: 16 h after inoculation, and Lane 3: 2 h after inoculation]. The green arrow indicates the expression of EGFP and the red arrow indicates the leaky expression of Hsp27, Hsp40, and Hsp70 on the gel a, b, and c, respectively. The protein marker also shows proteins with the molecular weights of 180, 135, 100, 75, 63, 48, 35, 25, 17, 11 kDa.

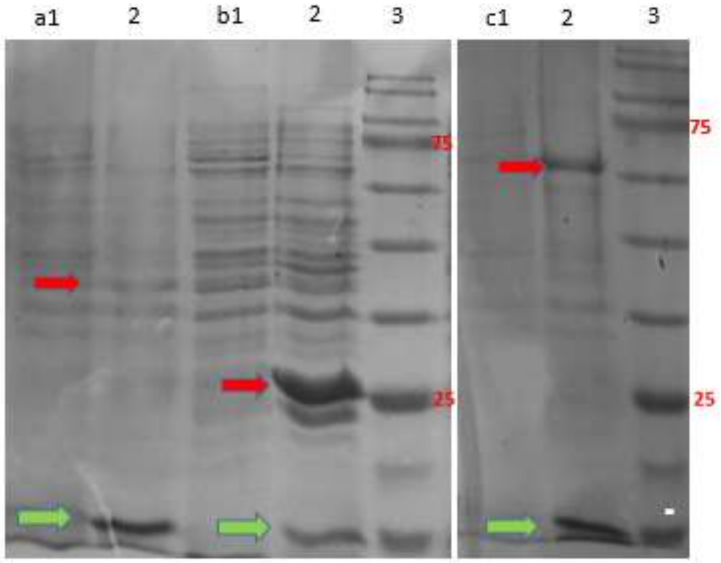

Figure S9. The SDS-PAGE analysis of Interleukin-2 expression in autoinducible systems. (**a**) Hsp40 SILEX system [lane 1: 2 h after inoculation, Lane 2: 16 h after inoculation], (**b**) Hsp27 SILEX system [lane 1: 2 h after inoculation, Lane 2: 16 h after inoculation, and lane 3: protein marker], (**c**) hsp70 SILEX system [lane 1: 2 h after inoculation, Lane 2: 16 h after inoculation, and lane 3: protein marker]. The green arrow indicates the expression of Interleukin-2 and the red arrow indicates the leaky expression of Hsp40, Hsp27, and Hsp70 on the gel a, b, and c,

respectively. The protein marker also shows proteins with the molecular weights of 180, 135, 100, 75, 63, 48, 35, 25, 17, 11 kDa.

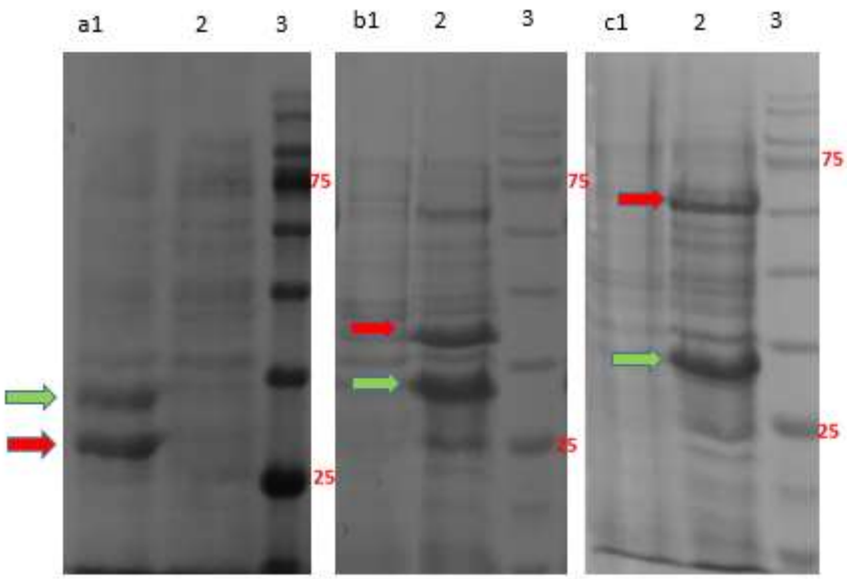

Figure S10. The SDS-PAGE analysis of romiplostim expression in autoinducible systems. (**a**) Hsp27 SILEX system [lane 1: 16 h after inoculation, Lane 2: 2 h after inoculation, and lane 3: protein marker], (**b**) Hsp40 SILEX system [lane 1: 2 h after inoculation, Lane 2: 16 h after inoculation, and lane 3: protein marker], (**c**) hsp70 SILEX system [lane 1: 2 h after inoculation, Lane 2: 16 h after inoculation, and lane 3: protein marker]. The green arrow indicates the expression of romiplostim and the red arrow indicates the leaky expression of Hsp27, Hsp40, and Hsp70 on the gel a, b, and c, respectively. The protein marker also shows proteins with the molecular weights of 180, 135, 100, 75, 63, 48, 35, 25, 17, 11 kDa.

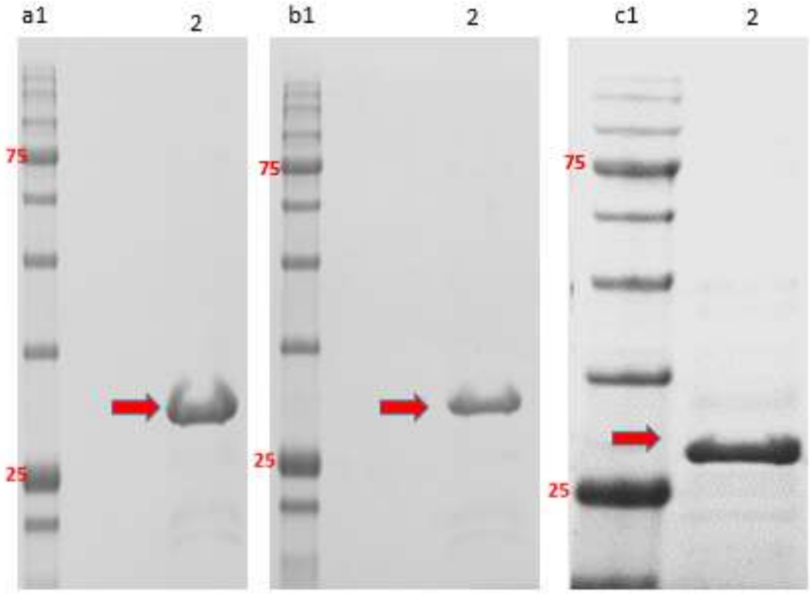

Figure S11. The SDS-PAGE analysis of purified EGFP. (**a**) Hsp27 SILEX system [Lane 1: protein marker, and Lane 2: purified EGFP], (**b**) Hsp40 SILEX system [Lane 1: protein marker, and Lane 2: purified EGFP], and (**c**) Hsp70 SILEX system [Lane 1: protein marker, and Lane 2: purified EGFP]. The red arrow indicates purified EGFP. The protein marker also shows proteins with the molecular weights of 180, 135, 100, 75, 63, 48, 35, 25, 17, 11 kDa.

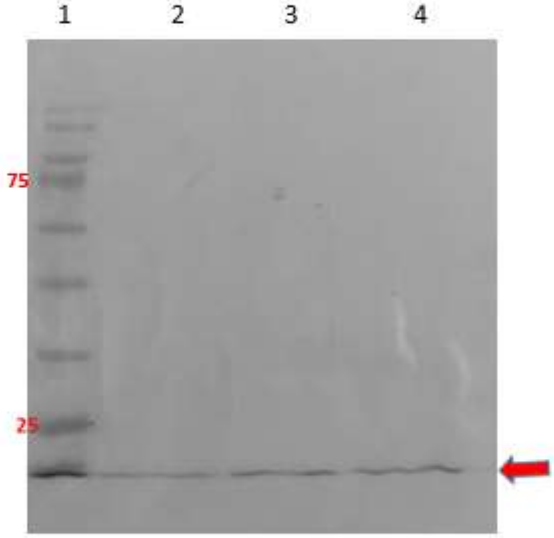

Figure 12. The SDS-PAGE analysis of purified interleukin-2 in autoinducible systems. Lane 1: protein marker, Lane 2: Hsp27 SILEX system, Lane 3; Hsp40 SILEX system, and Lane 4: Hsp70 SILEX system. The red arrow indicates purified interleukin-2. The protein marker also shows proteins with the molecular weights of 180, 135, 100, 75, 63, 48, 35, 25, 17, 11 kDa.

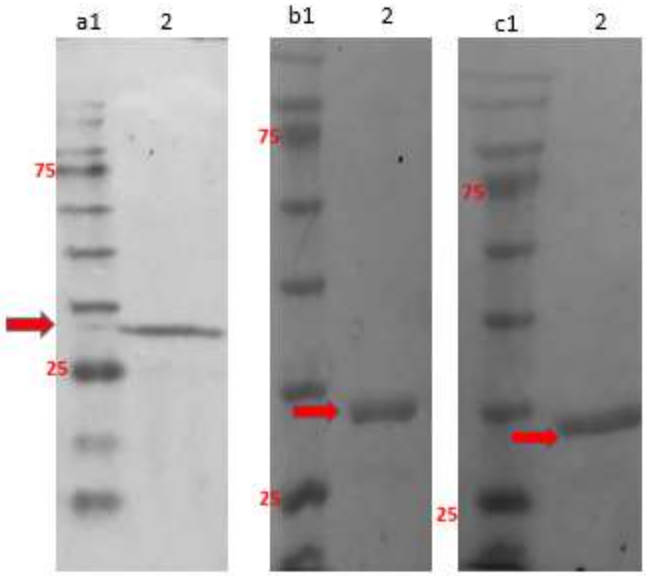

Figure 5. The SDS-PAGE analysis of purified romiplostim. (**a**) Hsp27 SILEX system [Lane 1: protein marker, and Lane 2: purified romiplostim], (**b**) Hsp40 SILEX system [Lane 1: protein marker, and Lane 2: purified romiplostim], and (**c**) Hsp70 SILEX system [Lane 1: protein marker, and Lane 2: purified romiplostim]. The red arrow indicates purified romiplostim. The protein marker also shows proteins with the molecular weights of 180, 135, 100, 75, 63, 48, 35, 25, 17, 11 kDa.

Table S1. The concentration of purified proteins in SILEX systems.

SILEX System	EGFP (mg/ml)	Romiplostim (mg/ml)	Interleukin (mg/ml)
**Hsp27**	0.75 ± 0.053	0.60 ± 0.012	0.50 ± 0.037
**Hsp40**	0. 47 ± 0.030	0.47 ± 0.028	0.47 ± 0.053
**Hsp70**	0.50 ± 0.010	0.49 ± 0.036	0.45 ± 0.020
